# Effect of Adjuvant Magnetic Fields in Radiotherapy on Non-Small-Cell Lung Cancer Cells *In Vitro*

**DOI:** 10.1155/2013/657259

**Published:** 2013-10-10

**Authors:** Jianguo Feng, Huaying Sheng, Chihong Zhu, Hao Jiang, Shenglin Ma

**Affiliations:** ^1^Cancer Research Institute, Zhejiang Cancer Hospital, No. 38 Guangji Road, Hangzhou, Zhejiang 310022, China; ^2^Key Laboratory Diagnosis and Treatment Technology on Thoracic Oncology, Hangzhou, Zhejiang 310022, China; ^3^Department of Oncology, Affiliated Hangzhou Hospital (Hangzhou First People's Hospital), Nanjing Medical University, No. 261 Huansha Road, Hangzhou 310006, China

## Abstract

*Objectives*. To explore sensitization and possible mechanisms of adjuvant magnetic fields (MFs) in radiotherapy (RT) of non-small-cell lung cancer. *Methods*. Human A549 lung adenocarcinoma cells were treated with MF, RT, and combined MF-RT. Colony-forming efficiency was calculated, cell cycle and apoptosis were measured, and changes in cell cycle- and apoptosis-related gene expression were measured by microarray. *Results*. A 0.5 T, 8 Hz stationary MF showed a duration-dependent inhibitory effect lasting for 1–4 hours. The MF-treated groups had significantly greater cell inhibition than did controls (*P* < 0.05). Surviving fractions and growth curves derived from colony-forming assay showed that the MF-only, RT-only, and MF-RT groups had inhibited cell growth; the MF-RT group showed a synergetic effect. Microarray of A549 cells exposed for 1 hour to MF showed that 19 cell cycle- and apoptosis-related genes had 2-fold upregulation and 40 genes had 2-fold downregulation. MF significantly arrested cells in G_2_ and M phases, apparently sensitizing the cells to RT. *Conclusions*. MF may inhibit A549 cells and can increase their sensitivity to RT, possibly by affecting cell cycle- and apoptosis-related signaling pathways.

## 1. Introduction

Lung cancer is a common malignant tumor, and its incidence is rapidly growing: 64% of patients with non-small-cell lung cancer (NSCLC) need radiotherapy (RT); 45% of these patients receive primary RT. Although RT and chemotherapy together have better therapeutic effects, patients often cannot tolerate the toxicity and side effects of the combination. Optimizing treatment result is therefore critical.

Magnetic fields (MFs) are biologically effective, and their effect on tumors has been studied since the 1970s [1–5]. Although the mechanism of how MFs affect tumors is unclear, they have been shown to inhibit cancer cell growth and induce apoptosis. Magnetic fields influence charged particles. As such, they interfere with interactions among molecules and electrons in cells and possibly harm cellular functions such as DNA synthesis, thereby inhibiting cancer cell division and growth [6]. Zhang et al. reported that a 3 Hz/picosecond electromagnetic pulse can apparently inhibit growth of cervical carcinoma Hela cells by raising intercellular Ca^2+^ concentration, inducing apoptosis, and increasing Bax protein expression while decreasing Bcl-2 expression (thus significantly increasing the Bax/Bcl-2 ratio) [7]. Lu et al. applied a low-frequency electromagnetic field on BEL-7402 hepatoma cells and found that expression of *SODD* and *Survivin* genes was significantly downregulated [8]. Wei et al. studied effects of rotational MFs combined with 5-fluorouracil (5-FU) on cell cycle and apoptosis in SP2/0 mouse myeloma cells, and found the S phase ratio was increased [9]. Magnetic fields alone cannot induce cell apoptosis, but they can sensitize cells to 5-FU toxicity, thus facilitating 5-FU-induced apoptosis. Liu et al. claimed that strong magnetic pulses significantly inhibited growth and exacerbated apoptosis in BIU-87 bladder carcinoma cells [10]. Pan et al. used microarray to measure and analyze the apoptosis-related gene-expression profile in MF-processed BEL-7402 hepatoma cells and L-02 fetus liver cells [11]. Electromagnetic field-processed cells upregulated expression of apoptosis-inducing genes and downregulated expression of apoptosis-inhibiting genes. Han et al. used pulse MFs to study drug resistance in HL60/ADR leukemia cells [12]. Pulse MFs could downregulate MRP1 gene and protein expression, while increasing accumulation of cellular Rg123, and reverse multidrug resistance in leukemia cells.

Preliminary research showed that MFs, alone or together with chemotherapy, can inhibit tumor cell proliferation. However, few studies of MFs combined with RT in lung cancer are reported. We hypothesized that cell-cycle changes induced by MFs sensitize lung cancer cells to radiation. In this study, we designed experiments to measure the effect of adjuvant MFs in chemotherapy on colony formation, cell cycle, and apoptosis in A549 cells. Microarray was employed to elucidate the molecular and cellular mechanisms. 

## 2. Materials and Methods

### 2.1. Cell Lines and Reagents

Lung adenocarcinoma cell line A549 was provided by Zhejiang Cancer Hospital. Cells were cultured in RPMI1640 media with 10% bovine serum and kept in an incubator at 5% CO_2_ and 37°C to promote growth. RPMI1640 was purchased from Gibco-BRL; bovine serum was purchased from HyClon.

### 2.2. Magnetic Field Duration and Radiation Dose

The inhibition rate was estimated by MTT assay to determine the duration of the MF effect. Using earlier research [13], 4 Gy was chosen as the radiation dose. Cells were transferred into 96-well plates at 500 cells/well and cultured for 24 hours. Four 8-well groups of cells were exposed to 0.5 T stationary MFs for 1, 2, 3, or 4 hours. After 48 hours, 20 *μ*L 5 mg/mL MTT was added into each well. After culturing for another 4 hours, supernatant was disposed, and 200 *μ*L was added into each well. After another 30 minutes, when brown crystals were completely dissolved, absorbance (AB) of each well was measured by enzyme-linked immunosorbent assay with 550 nm absorption wavelength. Inhibition rate of cell growth was calculated as [(Experimental AB − background control AB)/(Control AB − background control AB)] × 100%.

### 2.3. Colony-Forming and Surviving Curve Assay

Cells in logarithmic growth phase were digested into single-cell suspensions which were diluted and transferred into 6-well plates with 400 cells per well. After 24-hour adherent culturing, all cells were divided into 12 groups, each consisting of one plate of cells: one control group, five RT-only groups (2, 4, 6, 8, or 10 Gy), one MF-alone group (0.5 T, 8 Hz for 1 hour), and five MF-RT combination groups (0.5 T, 8 Hz for 1 hour; plus 2, 4, 6, 8, or 10 Gy). Colonies were counted after 10-hour culture. Colony-forming efficiency (CE) and surviving fraction (SF) were calculated with the following equations:

(1) $$\begin{array}{c} \text{CE}=\frac{\text{Colonies}\;\text{observed}}{\text{Number}\;\text{of}\;\text{cells}\;\text{plated}},\\ \text{SF}=\frac{\text{CE}\;\text{of}\;\text{treated}\;\text{group}}{\text{CE}\;\text{of}\;\text{control}\;\text{group}}. \end{array}$$

Survival curves were drawn using multitarget single-hit models and linear quadratic models with SigmaPlot 10.0 software.

### 2.4. Superarray Gene Chip Assay

Cells at logarithmic growth phase were digested into single-cell suspensions, which were diluted and transferred into 75 mL culture flasks with 1 × 10^5^ cells per flask. After 24-hour adherent culturing, three flasks of cells were exposed to 0.5 T, 8 Hz MF for 1 hour, and three bottles of cells were used as controls. After another 24 hours of culturing, RNA was extracted for gene chip assays for each group.

### 2.5. Cell Cycle and Apoptosis Assay

Cells in logarithmic growth phase were digested into single cell suspensions, which were diluted and transferred into 25 mL culture flasks with 5 × 10^4^ cells per flask. Cells were randomly divided into four groups: controls, MF-only group (0.5 T), RT-only group (4 Gy), and combination group (0.5 T + 4 Gy). Each group provided three parallel flasks for collection at 24, 48, and 72 hours separately. Cell cycle and apoptosis rates were measured by flow cytometry with an ABC cell cycle kit (BD Biosciences) and an Annexin V-FITC apoptosis detection kit.

### 2.6. Data Analysis

SPSS 11.0 software was used for statistical analysis. Measurement data are expressed as mean ± standard deviation. Different groups were compared using one-way ANOVA. *P* < 0.05 was considered statistically significant.

## 3. Results 

### 3.1. Inhibition Rates under Different Magnetic Field Durations Measured with MTT Assay

The inhibitory effect of a 0.5 T, 8 Hz stationary MF lasts for 1–4 hours, in a duration-dependent manner (Table 1). Although the inhibitory effect did not significantly differ with magnetic duration (*P* > 0.05), MF-treated groups had significantly greater cell inhibition than the control group (*P* < 0.05). 

### 3.2. Colony-Forming Efficiency and Surviving Curve

**Figure 1:**
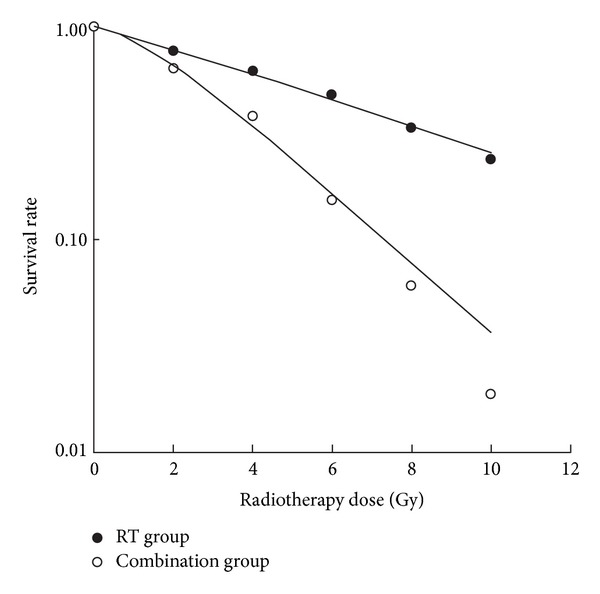
Cell survival rate after treatment with different dose of radiotherapy.

The colony-forming assay showed that, for RT-only groups at 2, 4, 6, 8, and 10 Gy, the CEs were 16.4%, 13.2%, 10.2%, 7.1%, and 1.2%, respectively; SFs were 0.77, 0.62, 0.48, 0.33, and 0.24, respectively. For MF-RT combined groups at 2, 4, 6, 8, and 10 Gy, CEs were 13.7%, 8.1%, 3.3%, 1.3%, and 0.4%, respectively, and SFs were 0.64, 0.38, 0.15, 0.06, and 0.02, respectively. Cell survival decreased significantly (*P* < 0.05) with increasing RT dose in both RT groups and combination groups. Among groups with the same RT dose, the group with adjuvant MFs had a significantly smaller SF (*P* < 0.05), which suggests that A549 cells are more sensitive to RT with adjuvant MFs application. Survival curves are shown in Figure 1.

### 3.3. Gene Chip Assay

The microarray showed that after 1-hour exposure to MFs, 19 cell cycle- and apoptosis-related genes in the A549 cells had 2-fold upregulation, and 40 genes had 2-fold downregulation (Tables 2 and 3). In particular, *TNFRSF21* and CASPASE had significant upregulation, whereas expressions of *ATM*, *p53*, *p57*, *p21*, *p27*, *TNFSF12*, *TNFRSF10D*, *BAG4*, *BCL2L2*, *Mdn2*, and *XRCC1–5* were downregulated.

### 3.4. The Alternation of Cell Cycle and Apoptosis

**Figure 2:**
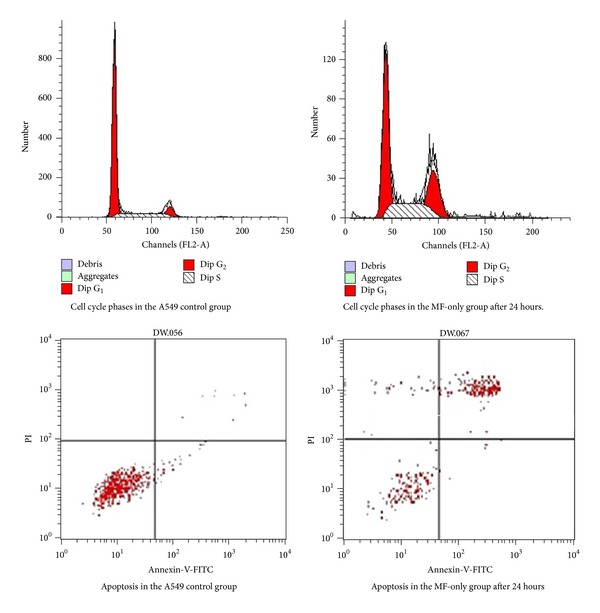
Changes in cell cycle and apoptosis.

Flow cytometry results showed that the MF-only group had G_2_-M phase arrest. Percentages of MF-only cells at G_2_-M were 24.2% for collection at 24 hours, 28.4% at 48, hours and 18.5% at 72 hours—all significantly different from the control group. The MF-only group showed no significant difference in apoptosis index compared with the control group. Both the RT-only group and the MF-RT combination group showed significant apoptosis; however, the apoptosis index of combination group was 34.6 for collection after 24 hours, which was significantly higher than that of the RT-only group (Figure 2).

## 4. Discussion

Repair of DNA double-strand breaks (DSBs) and cell-cycle regulation are two important factors that influence RT sensitivity of cells. ATM plays a very important role in DSB repair and cell cycle regulation signaling pathways. ATM activates the G_1_-S checkpoint by activating p53 and p21 genes; it activates S phase and G_2_-M checkpoints by activating the chk1, chk2, cdc25, and cdc2 genes [14]. When ATM expression is deficient or decreased, cell cycle checkpoints are dysfunctional, and cell cycle arrest is hindered. Thus, ATM expression and activity are related to RT sensitivity of cells [15]. In a study of sensitivity of nasopharyngeal carcinoma cell CNE-1 to RT, Hui et al. found that an RT sensitizing agent, UCN-01, works by weakening the cell's self-repair capability, and UCN-01 can only sensitize cells with *p53* deficiency. Cyclin-dependent kinase inhibitor 1C (CDKN1C; p57, Kip2), which belongs to Cip/Kip family, can inhibit multiple G_1_ cyclin/Cdk complexes and induce G_1_ arrest, thus inhibiting cell proliferation. CDKN1A (p21, Cip1) can inhibit CDK2 or CDK4 complexes and regulate the cell cycle. CDKN1A is regulated by p53 and can arrest cell in G_1_ phase under activating circumstances. CDKN1B (p27, Kip1), which encodes a CDK-inhibitor protein, can inhibit activation of cyclin E/CDK2 or cyclin D/CDK4 complexes and arrest the G_1_ phase as well. TNFSF12, which belongs to TNF superfamily, can combine with the FN14/TWEAKR cytokine receptor, thus inducing apoptosis through multiple cell death pathways, and promote endothelial cell proliferation and migration (which are related to angiogenesis). TNFRSF21, whose functional domain activates the NF-*κ*B and MAPK8/JNK pathways, also induces apoptosis. However, TNFRSF10D does not induce apoptosis and has been shown to play an inhibitory role in TRAIL-induced cell apoptosis. BAG4 is a member of the BAG1-related protein family. BAG4 is an antiapoptosis protein; it can interact with multiple apoptosis- and cell growth-related proteins, including BCL-2, Raf kinase, steroid receptor, growth factor receptor, and heat shock protein; it combines with TNFR1 and death receptor 3 to negatively regulate the downstream death signaling pathway. BCL2L2 belongs to the bcl-2 family; its expression induces apoptosis under cellular toxic environment. Mdn2 protein combines with and deactivates p53 and RB proteins, and it negatively regulates the *p53* gene. X-ray repair cross-complementing gene (*XRCC*) is a major mediator of mammalian gene repair [16]. XRCC1, XPD, and XRCC3 proteins are the important components of BER, NER, and DSBR, respectively. XRCC1 repairs DNA single-strand breaks, induced by RT or alkylation agents, and works with DNA ligase III, polymerase beta, and poly(ADP-ribose) polymerase, involved in the BER pathway. XRCC2 and XRCC3 mediate RecA/Rad51-related proteins involved in homologous recombination to maintain chromosome stability and repair of double-strand breaks in DNA damage.

The gene chip results showed that, after MF exposure of A549 cells, the apoptosis-inducing gene TNFRSF21 was upregulated, as were several other apoptosis-related genes (e.g., ATM, p53, p57, p21, p27, TNFSF12, TNFRSF10D, BAG4, BCL2L2, Mdn2, and XRCC1–5). The upregulation of TNFRSF21 activated NF-*κ*B and APK8/JNK pathways and induced apoptosis. Cellular sensitivity to RT is related to apoptosis rate [17]; higher apoptosis levels indicate higher sensitivity to RT, and rapidly apoptotic cells are more sensitive to RT. Conversely, downregulation of ATM and p53 increases apoptosis; downregulation of p57, p21, and p27 weakens cell-cycle arresting function, thus inducing apoptosis; downregulation of antiapoptotic genes (TNFSF12, TNFRSF10D, BAG4, BCL2L2, and Mdn2) also induces apoptosis. Downregulation of XRCC1–5 also weakens DNA repair function, thus leading to cell death and weakened proliferative capacity.

Our study showed that, for a 0.5 T, 8 Hz stationary MF, duration had no significant effect (*P* > 0.05); however, groups treated with MF had significantly greater cell inhibition than controls (*P* > 0.05). The surviving fraction and growth curve derived from the colony-forming assay showed that MF-only, 4 Gy RT-only and the MF-RT combination groups had inhibited cell growth; the combination group in particular showed a synergetic effect (*P* > 0.01). The microarray showed that after A549 cells were exposed for 1 hour to MFs, 19 cell cycle- and apoptosis-related genes had 2-fold upregulation, especially TNFRSF21 and CASPASE, and 40 genes, including ATM, p53, p57, p21, p27, TNFSF12, TNFRSF10D, BAG4, BCL2L2, Mdn2, and XRCC1–5, had 2-fold downregulation. Magnetic fields significantly arrested cells in the G_2_ and M phases, which are the RT-sensitive phases; in this case, the cells were sensitized to RT. This study explored this sensitization effect and possible mechanisms of adjuvant MFs with RT on NSCLC at cellular and gene levels. Further study is needed to further clarify these mechanisms.

## Figures and Tables

**Table 1:** Inhibition rates under different magnetic field durations.

Magnetic field duration (h)	Absorbance (OD value)	Inhibition rate (%)
Control	1.120 ± 0.089	0.0
1 h	1.032 ± 0.059	7.9
2 h	1.025 ± 0.065	8.5
3 h	0.990 ± 0.087	11.6
4 h	0.985 ± 0.098	12.1

**Table 2:** Upregulated genes in A549 after 1-hour exposure to MF.

Position	Genebank	Gene name	Fold change
138	NM_003824	FADD	5.64
278	NM_006297	XRCC1	4.32
89	NM_001260	CDK8	3.42
225	NM_003839	Rank	3.11
199	NM_000963	Cox-2	2.87
261	NM_003300	CRAF1	2.81
236	NM_000043	Fas/Apo-1/CD95	2.56
227	NM_003790	DR3/Apo3	2.52
63	NM_053056	Cyclin D1	2.48
62	NM_005190	Cyclin C	2.45
244	NM_003809	TNFSF12/APO3L	2.37
43	NM_003723	Caspase 13	2.26
44	NM_012114	Caspase 14	2.14
58	NM_003914	Cyclin A1	2.12
182	NM_002392	Mdm2	2.08
61	NM_004701	Cyclin B2	2.08
70	NM_004354	Cyclin G2	2.05
48	NM_004347	Caspase-5	2.03
64	NM_001759	Cyclin D2	2.00

**Table 3:** Downregulated genes in A549 after 1-hour exposure to MF.

Position	Genebank	Gene name	Fold change
228	NM_003820	TNFRSF14	0.03
246	NM_006573	TNFSF13B	0.09
264	NM_004620	TRAF6	0.12
96	NM_001800	p19-INK4D	0.14
232	NM_001066	TNFR2/p75	0.15
86	NM_000075	Cdk4	0.17
90	NM_000389	P21/Waf1/CIP1	0.18
221	NM_003844	TRAIL-R/DR4	0.20
88	NM_001799	CDK7	0.21
128	NM_001950	E2F-4	0.21
229	NM_001192	TNFRSF17	0.22
76	NM_004358	CDC25B	0.23
127	NM_001949	E2F-3	0.23
282	NM_021141	KU80	0.26
130	NM_001952	E2F-6	0.27
135	NM_005236	XPF	0.33
204	NM_000321	Rb	0.34
114	NM_000499	CYP1A1	0.34
262	NM_004295	TRAF-4	0.35
122	NM_004402	DFF40/CPAN	0.36
26	NM_004050	Bcl-w	0.38
279	NM_005431	XRCC2	0.41
7	NM_000051	ATM	0.41
17	NM_001188	Bak	0.41
132	NM_001983	ERCC1	0.42
22	NM_000633	Bcl-2	0.42
208	NM_003804	RIP	0.43
126	NM_004091	E2F-2	0.43
142	NM_001924	GADD45	0.44
91	NM_004064	p27Kip1	0.44
71	NM_001239	Cyclin H	0.45
27	NM_005178	BCL-3	0.45
281	NM_003401	XRCC4	0.46
256	NM_000546	p53	0.46
129	NM_001951	E2F-5	0.48
242	NM_003810	TRAIL	0.48
125	NM_005225	E2F	0.48
240	NM_001561	4-1BB	0.49
224	NM_003840	TRAIL-R4/DcR2	0.49
220	NM_000594	TNFA	0.50
